# 2-Isopropyl-3-methyl­quinoxaline 1,4-dioxide

**DOI:** 10.1107/S1600536810023706

**Published:** 2010-07-03

**Authors:** Jian-Ye Li, Tao Sun, Ai-You Hao, Hongwei Qiao, Feifei Xin

**Affiliations:** a School of Chemistry and Chemical Engineering, Shandong University, Jinan 250100, People’s Republic of China; b Shandong Shengquan Chemical Co. Ltd, Jinan 250204, People’s Republic of China

## Abstract

In the title compound, C_12_H_14_N_2_O_2_, the quinoxaline ring system and the C atoms of the methylene and methyl substituents lie on a mirror plane. The crystal packing is stabilized by weak π–π inter­actions [centroid–centroid distance = 3.680 (7) Å].

## Related literature

For the preparation, see: Issidorides & Haddadin (1966 ▶). For the biological activity of quinoxaline di-*N*-oxide compounds, see: Amin *et al.* (2006 ▶); Edwards *et al.* (1975 ▶); Glazer & Chappel (1982 ▶).
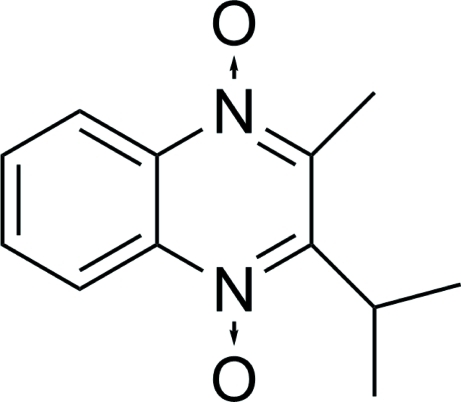


## Experimental

### 

#### Crystal data

C_12_H_14_N_2_O_2_
*M* *_r_* = 218.25
Orthorhombic, 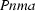

*a* = 13.3879 (10) Å
*b* = 6.8462 (6) Å
*c* = 11.8861 (9) Å
*V* = 1089.44 (15) Å^3^
*Z* = 4
Mo *K*α radiation
μ = 0.09 mm^−1^
*T* = 296 K
0.29 × 0.27 × 0.26 mm

#### Data collection

Bruker APEXII CCD area-detector diffractometer
Absorption correction: multi-scan (*SADABS*; Bruker, 2005 ▶) *T*_min_ = 0.651, *T*_max_ = 0.745
10376 measured reflections
1446 independent reflections
1062 reflections with *I* > 2σ(*I*)
*R*_int_ = 0.019

#### Refinement

*R*[*F* ^2^ > 2σ(*F* ^2^)] = 0.057
*wR*(*F* ^2^) = 0.203
*S* = 1.17
1446 reflections
96 parameters
1 restraint
H-atom parameters constrained
Δρ_max_ = 0.38 e Å^−3^
Δρ_min_ = −0.35 e Å^−3^

### 

Data collection: *APEX2* (Bruker, 2005 ▶); cell refinement: *SAINT* (Bruker, 2005 ▶); data reduction: *SAINT*; program(s) used to solve structure: *SIR97* (Altomare *et al.*, 1999 ▶); program(s) used to refine structure: *SHELXL97* (Sheldrick, 2008 ▶); molecular graphics: *SHELXTL* (Sheldrick, 2008 ▶); software used to prepare material for publication: *WinGX* (Farrugia, 1999 ▶).

## Supplementary Material

Crystal structure: contains datablocks I, global. DOI: 10.1107/S1600536810023706/jh2164sup1.cif

Structure factors: contains datablocks I. DOI: 10.1107/S1600536810023706/jh2164Isup2.hkl

Additional supplementary materials:  crystallographic information; 3D view; checkCIF report

## Figures and Tables

**Fig. 1.**
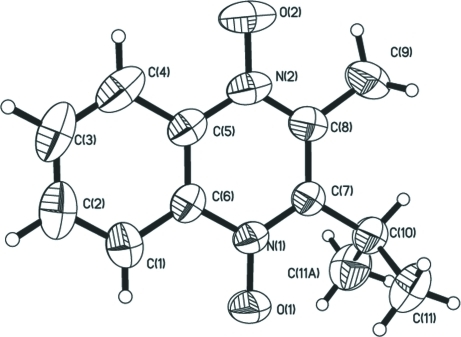
Ellipsoid plot.

**Fig. 2.**
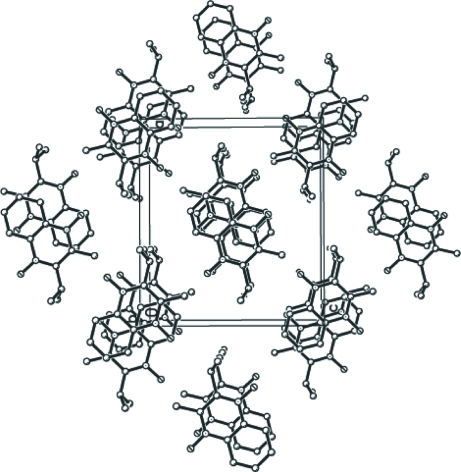
Packing diagram.

## supplementary crystallographic information

### Comment

In recent years, the compounds of quinoxaline di-N-oxide have been used as important and widely-used drugs for sterilization and growth-promoting of animals. Quinoxaline di-N-oxide also has pharmacological properties usable as intermediates for producing plant protection agents. The research in the crystal of quinoxaline di-N-oxide has great meaning to pharmacology. The title compound 2-isopropyl-3-methylquinoxaline 1,4-dioxide was obtained by Beirut Reaction: benzofurazan-N-oxides reacted with cyclohexanone catalysed by triethylamine without any other solvent.

### Experimental

Yellow crystals were obtained by slow evaporation of the solvents from solutions of the title compound in methanol. ^1^H NMR (400 MHz, DMSO-d6): δ 8.43 (2*H*, d, J = 3.5 Hz, Ar—H), 7.88 (2*H*, d, J = 3.2 Hz, Ar—H), 2.93 (4*H*, s, CH_2_), 1.83 (4*H*, s, CH_2_); IRνmax (KBr, cm^-1^): 3455, 3125, 2943, 2866, 1987, 1953, 1737, 1605, 1516, 1441, 1422, 1400, 1357, 1315, 1277, 1246, 1125, 1089, 1016, 979, 933, 904, 842, 824, 776, 694, 668, 640, 613, 557, 528, 436; Calcd for C_12_H_12_N_2_O_2_: C, 66.65; H, 5.59; N, 12.96. Found: C, 66.34; H, 5.32; N, 12.90; ESIMS calcd for C_12_H_12_N_2_O_2_H^+^ m/*z* 217.24, found m/*z* 217.20.

### Refinement

Refinement of *F*^2^ against ALL reflections. The weighted *R*-factor *wR* and goodness of fit S are based on *F*^2^, conventional *R*-factors *R* are based on F, with F set to zero for negative*F*^2^. The threshold expression of *F*^2^ > 2sigma(*F*^2^) is used only for calculating *R*-factors(gt) *etc*. and is not relevant to the choice of reflections for refinement. *R*-factors based on *F*^2^ are statistically about twice as large as those based on F, and R– factors based on ALL data will be even larger.

### Crystal data

**Table:**

C_12_H_14_N_2_O_2_	*D*_x_ = 1.331 Mg m^−^^3^
*M**_r_* = 218.25	Mo *K*α radiation, λ = 0.71073 Å
Orthorhombic, *P**n**m**a*	Cell parameters from 4638 reflections
*a* = 13.3879 (10) Å	θ = 3.0–27.9°
*b* = 6.8462 (6) Å	µ = 0.09 mm^−^^1^
*c* = 11.8861 (9) Å	*T* = 296 K
*V* = 1089.44 (15) Å^3^	Block, colourless
*Z* = 4	0.29 × 0.27 × 0.26 mm
*F*(000) = 464	

### Data collection

**Table:**

Bruker APEXII CCD area-detector diffractometer	1446 independent reflections
Radiation source: fine-focus sealed tube	1062 reflections with *I* > 2σ(*I*)
graphite	*R*_int_ = 0.019
φ and ω scans	θ_max_ = 28.6°, θ_min_ = 2.3°
Absorption correction: multi-scan (*SADABS*; Bruker, 2005)	*h* = −18→17
*T*_min_ = 0.651, *T*_max_ = 0.745	*k* = −9→9
10376 measured reflections	*l* = −16→16

### Refinement

**Table:**

Refinement on *F*^2^	Secondary atom site location: difference Fourier map
Least-squares matrix: full	Hydrogen site location: inferred from neighbouring sites
*R*[*F*^2^ > 2σ(*F*^2^)] = 0.057	H-atom parameters constrained
*wR*(*F*^2^) = 0.203	*w* = 1/[σ^2^(*F*_o_^2^) + (0.0863*P*)^2^ + 0.4481*P*] where *P* = (*F*_o_^2^ + 2*F*_c_^2^)/3
*S* = 1.17	(Δ/σ)_max_ < 0.001
1446 reflections	Δρ_max_ = 0.38 e Å^−^^3^
96 parameters	Δρ_min_ = −0.35 e Å^−^^3^
1 restraint	Extinction correction: *SHELXL97* (Sheldrick, 2008), Fc^*^=kFc[1+0.001xFc^2^λ^3^/sin(2θ)]^-1/4^
Primary atom site location: structure-invariant direct methods	Extinction coefficient: 0.011 (4)

### Special details

**Table:**

Geometry. All e.s.d.'s (except the e.s.d. in the dihedral angle between two l.s. planes) are estimated using the full covariance matrix. The cell e.s.d.'s are taken into account individually in the estimation of e.s.d.'s in distances, angles and torsion angles; correlations between e.s.d.'s in cell parameters are only used when they are defined by crystal symmetry. An approximate (isotropic) treatment of cell e.s.d.'s is used for estimating e.s.d.'s involving l.s. planes.
Refinement. Refinement of *F*^2^ against ALL reflections. The weighted *R*-factor *wR* and goodness of fit *S* are based on *F*^2^, conventional *R*-factors *R* are based on *F*, with *F* set to zero for negative *F*^2^. The threshold expression of *F*^2^ > σ(*F*^2^) is used only for calculating *R*-factors(gt) *etc*. and is not relevant to the choice of reflections for refinement. *R*-factors based on *F*^2^ are statistically about twice as large as those based on *F*, and *R*- factors based on ALL data will be even larger.

### Fractional atomic coordinates and isotropic or equivalent isotropic displacement parameters (Å^2^)

**Table:**

	*x*	*y*	*z*	*U*_iso_*/*U*_eq_	
C1	0.9509 (2)	0.2500	1.1853 (3)	0.0603 (8)	
H11	0.9026	0.2500	1.2416	0.072*	
C2	1.0499 (3)	0.2500	1.2128 (4)	0.0731 (10)	
H2	1.0689	0.2500	1.2880	0.088*	
C3	1.1212 (2)	0.2500	1.1306 (4)	0.0723 (11)	
H3	1.1883	0.2500	1.1510	0.087*	
C4	1.0962 (2)	0.2500	1.0186 (4)	0.0641 (9)	
H4	1.1454	0.2500	0.9633	0.077*	
C5	0.99369 (19)	0.2500	0.9893 (2)	0.0449 (6)	
C6	0.92245 (18)	0.2500	1.0730 (2)	0.0440 (6)	
C7	0.79331 (18)	0.2500	0.9352 (2)	0.0427 (6)	
C8	0.8664 (2)	0.2500	0.8500 (2)	0.0481 (6)	
C9	0.8419 (3)	0.2500	0.7287 (3)	0.0705 (9)	
H9A	0.9023	0.2500	0.6845	0.080*	
H9B	0.8034	0.1355	0.7099	0.080*	
C10	0.6836 (2)	0.2500	0.9063 (3)	0.0551 (7)	
H10	0.6798	0.2500	0.8240	0.066*	
C11	0.63084 (17)	0.0655 (4)	0.9452 (2)	0.0777 (8)	
H11A	0.6657	−0.0468	0.9169	0.117*	
H11B	0.5636	0.0652	0.9173	0.117*	
H11C	0.6299	0.0615	1.0259	0.117*	
N1	0.82094 (15)	0.2500	1.04424 (19)	0.0445 (6)	
N2	0.96482 (17)	0.2500	0.8772 (2)	0.0513 (6)	
O1	0.75580 (15)	0.2500	1.12490 (18)	0.0681 (7)	
O2	1.03357 (18)	0.2500	0.8002 (2)	0.0809 (8)	

### Atomic displacement parameters (Å^2^)

**Table:**

	*U*^11^	*U*^22^	*U*^33^	*U*^12^	*U*^13^	*U*^23^
C1	0.0617 (17)	0.0652 (18)	0.0539 (18)	0.000	−0.0125 (14)	0.000
C2	0.067 (2)	0.066 (2)	0.085 (3)	0.000	−0.0309 (19)	0.000
C3	0.0494 (16)	0.0531 (16)	0.114 (3)	0.000	−0.0284 (19)	0.000
C4	0.0403 (13)	0.0433 (14)	0.109 (3)	0.000	0.0089 (16)	0.000
C5	0.0405 (12)	0.0326 (11)	0.0616 (17)	0.000	0.0047 (11)	0.000
C6	0.0389 (12)	0.0377 (12)	0.0553 (15)	0.000	−0.0038 (11)	0.000
C7	0.0408 (12)	0.0438 (12)	0.0434 (14)	0.000	−0.0006 (10)	0.000
C8	0.0557 (14)	0.0409 (12)	0.0477 (15)	0.000	0.0076 (12)	0.000
C9	0.084 (2)	0.081 (2)	0.0466 (17)	0.000	0.0075 (16)	0.000
C10	0.0409 (13)	0.0706 (18)	0.0537 (17)	0.000	−0.0040 (12)	0.000
C11	0.0525 (11)	0.0818 (17)	0.099 (2)	−0.0170 (11)	−0.0097 (12)	0.0027 (15)
N1	0.0368 (10)	0.0509 (12)	0.0457 (12)	0.000	0.0050 (9)	0.000
N2	0.0485 (12)	0.0406 (11)	0.0648 (15)	0.000	0.0196 (11)	0.000
O1	0.0461 (10)	0.1067 (18)	0.0516 (12)	0.000	0.0126 (9)	0.000
O2	0.0716 (15)	0.0876 (17)	0.0836 (18)	0.000	0.0422 (13)	0.000

### Geometric parameters (Å, °)

**Table:**

C1—C2	1.365 (4)	C7—C10	1.508 (4)
C1—C6	1.389 (4)	C8—N2	1.357 (4)
C1—H11	0.9300	C8—C9	1.479 (4)
C2—C3	1.366 (6)	C9—H9A	0.964 (4)
C2—H2	0.9300	C9—H9B	0.964 (2)
C3—C4	1.373 (5)	C10—C11^i^	1.519 (3)
C3—H3	0.9300	C10—C11	1.519 (3)
C4—C5	1.416 (4)	C10—H10	0.9800
C4—H4	0.9300	C11—H11A	0.9600
C5—C6	1.378 (4)	C11—H11B	0.9600
C5—N2	1.387 (4)	C11—H11C	0.9600
C6—N1	1.401 (3)	N1—O1	1.296 (3)
C7—N1	1.348 (3)	N2—O2	1.298 (3)
C7—C8	1.409 (4)		
			
C2—C1—C6	119.7 (3)	C7—C8—C9	123.1 (3)
C2—C1—H11	120.1	C8—C9—H9A	110.2 (3)
C6—C1—H11	120.1	C8—C9—H9B	110.1 (2)
C1—C2—C3	120.5 (3)	H9A—C9—H9B	108.8 (2)
C1—C2—H2	119.7	C7—C10—C11^i^	112.56 (16)
C3—C2—H2	119.7	C7—C10—C11	112.56 (16)
C2—C3—C4	121.5 (3)	C11^i^—C10—C11	112.5 (3)
C2—C3—H3	119.2	C7—C10—H10	106.2
C4—C3—H3	119.2	C11^i^—C10—H10	106.2
C3—C4—C5	118.4 (3)	C11—C10—H10	106.2
C3—C4—H4	120.8	C10—C11—H11A	109.5
C5—C4—H4	120.8	C10—C11—H11B	109.5
C6—C5—N2	120.0 (2)	H11A—C11—H11B	109.5
C6—C5—C4	119.6 (3)	C10—C11—H11C	109.5
N2—C5—C4	120.4 (3)	H11A—C11—H11C	109.5
C5—C6—C1	120.3 (3)	H11B—C11—H11C	109.5
C5—C6—N1	119.7 (3)	O1—N1—C7	121.8 (2)
C1—C6—N1	120.0 (3)	O1—N1—C6	118.2 (2)
N1—C7—C8	120.0 (2)	C7—N1—C6	120.0 (2)
N1—C7—C10	119.1 (2)	O2—N2—C8	121.4 (3)
C8—C7—C10	120.9 (3)	O2—N2—C5	118.7 (2)
N2—C8—C7	120.2 (3)	C8—N2—C5	120.0 (2)
N2—C8—C9	116.6 (3)		
			
C6—C1—C2—C3	0.000 (2)	C8—C7—C10—C11	115.8 (2)
C1—C2—C3—C4	0.000 (2)	C8—C7—N1—O1	180.0
C2—C3—C4—C5	0.000 (1)	C10—C7—N1—O1	0.0
C3—C4—C5—C6	0.000 (1)	C8—C7—N1—C6	0.0
C3—C4—C5—N2	180.000 (1)	C10—C7—N1—C6	180.0
N2—C5—C6—C1	180.0	C5—C6—N1—O1	180.0
C4—C5—C6—C1	0.000 (1)	C1—C6—N1—O1	0.0
N2—C5—C6—N1	0.0	C5—C6—N1—C7	0.0
C4—C5—C6—N1	180.0	C1—C6—N1—C7	180.0
C2—C1—C6—C5	0.000 (1)	C7—C8—N2—O2	180.0
C2—C1—C6—N1	180.0	C9—C8—N2—O2	0.0
N1—C7—C8—N2	0.0	C7—C8—N2—C5	0.0
C10—C7—C8—N2	180.0	C9—C8—N2—C5	180.0
N1—C7—C8—C9	180.0	C6—C5—N2—O2	180.0
C10—C7—C8—C9	0.0	C4—C5—N2—O2	0.0
N1—C7—C10—C11^i^	64.2 (2)	C6—C5—N2—C8	0.0
C8—C7—C10—C11^i^	−115.8 (2)	C4—C5—N2—C8	180.0
N1—C7—C10—C11	−64.2 (2)		

Symmetry codes: (i) *x*, −*y*+1/2, *z*.
